# Monoclonal Anti-PCSK9 Antibodies: Real-World Data

**DOI:** 10.3390/jcm13154543

**Published:** 2024-08-03

**Authors:** Giulia Guidotti, Viola Liberati, Andrea Sorrentino, Elena Lotti, Felice Crudele, Angela Rogolino, Aniello Sammartino, Margherita Slanzi, Anna Maria Gori, Rossella Marcucci, Martina Berteotti

**Affiliations:** 1 Department of Experimental and Clinical Medicine, University of Florence, 50134 Florence, Italy; giulia.guidotti@unifi.it (G.G.); viola.liberati@unifi.it (V.L.); aniello.sammartino@unifi.it (A.S.); margherita.slanzi@edu.unifi.it (M.S.); annamaria.gori@unifi.it (A.M.G.); martina.berteotti@unifi.it (M.B.); 2 Atherothrombotic Diseases, “Careggi” University Hospital, 50134 Florence, Italy; lottie@aou-careggi.toscana.it (E.L.); felice.crudele@unifi.it (F.C.); rogolinoa@aou-careggi.toscana.it (A.R.)

**Keywords:** PCSK9 monoclonal antibodies, real-world data, hypercholesterolemia, high-risk patients, female gender, HeFH

## Abstract

**Background:** Real-world data on the use of lipid-lowering therapy (LLT) in clinical practice show that about 80% of (very) high-cardiovascular (CV)-risk patients disregard the 2019 European Society of Cardiology (ESC) Guidelines’ recommendations on dyslipidemias. The availability of proprotein convertase subtilisin/kexin type 9 monoclonal antibodies (PCSK9mAb) should reduce this gap. Our aim was to provide data on PCSK9mAb use in clinical practice, investigating the achievement of the ESC Guidelines’ recommendations in the real world. **Methods:** Between April 2018 and December 2022, patients who started on PCSK9mAb therapy (140 mg of evolocumab or 75 mg or 150 mg of alirocumab, subcutaneous injection every 2 weeks) were included in a prospective registry. Our cohort consisted of 256 patients: 95 (37.1%) were women (mean age: 65.43 ± 11.12 yrs), 53 (20.7%) were at high CV risk, and 203 (79.3%) were at very high CV risk. **Results:** After one year of PCSK9mAb treatment, nearly 60% of patients demonstrated full adherence to the ESC Guidelines’ recommendations, defined as achieving at least a 50% reduction in low-density lipoprotein cholesterol (LDL-C) levels along with reaching LDL-C target levels (≤55 and ≤70 mg/dL for very high and high risk, respectively). Concomitant high-dose statin therapy emerged as the primary predictor of LDL-C target attainment. Heterozygous familial hypercholesterolemia (HeFH), statin intolerance, and female gender were associated with a significant lower probability of achieving LDL-C target levels. **Conclusions:** Our analysis confirms that PCSK9mAb treatment is safe and effective, enabling 60% of our cohort to fully achieve the LDL-C guideline recommendations. The use of high-intensity statins emerged as a significant predictor of efficacy. Conversely, familial hypercholesterolemia and female gender were identified as predictors of therapeutic failure. Hence, it is crucial to address disparities in cardiovascular disease prevention between genders and to enhance strategies for managing elevated LDL-C in HeFH patients.

## 1. Introduction

Atherosclerotic cardiovascular disease (ASCVD) is the leading cause of morbidity and mortality worldwide [1]. LDL cholesterol (LDL-C) is one of the main modifiable cardiovascular risk (CV) factors, since the accumulation of LDL-C and other apo-B-containing lipoproteins is a key event in the initiation and progression of the atherosclerotic process [2,3].

There is a continuous positive relation between ASCVD risk and blood LDL-C concentrations: the greater the cumulative lifelong exposure to LDL-C, the higher the CV risk. Conversely, larger reductions in LDL-C may result in larger reductions in risk, with no evidence of a threshold [2,4,5].

As a result, target levels of LDL-C have been progressively reduced over the years to the very ambitious targets recommended by the current guidelines [6,7]. However, these new targets have substantially amplified the gap between international guidelines’ recommendations and the LDL-C levels achieved in clinical practice, particularly in high- and very-high-CV-risk patients. Indeed, real-word data indicate that the percentage of patients at (very) high CV risk who achieve optimal LDL-C values is on average less than 20% [8], underscoring the need for polypharmacological treatment and the use of effective and well-tolerated drugs to reach the new targets [8,9,10,11,12].

Among the available therapeutic strategies, proprotein convertase subtilisin/kexin type 9 monoclonal antibodies (PCSK9mAb) are recommended in secondary prevention for patients at very high CV risk and in primary prevention for high-CV-risk patients with heterozygous familial hypercholesterolemia (HeFH), diabetes, or renal insufficiency not achieving the LDL-C goal while receiving a maximally tolerated dose of statins and ezetimibe [13]. Trials have demonstrated that PCSK9mAb (evolocumab and alirocumab) can achieve a 55–60% reduction in LDL-C, resulting in improved cardiovascular prognosis and reduced mortality [14,15]. Additionally, these drugs have a favorable safety profile and a convenient route of administration, leading to increased patient compliance and therapeutic adherence [16,17,18,19,20,21,22,23]. Therefore, we retrospectively analyzed data from patients on PCSK9mAb therapy in order to investigate the portion of (very) high-CV-risk patients who reached recommended LDL-C values and assess their adherence to the 2019 ESC Guidelines’ recommendations on dyslipidemias [7]. Indeed, the rate at which (very) high-CV-risk patients achieve recommended targets with these treatments in real-world settings remains unclear. Another aim of our study was to identify potential independent predictors of LDL-C target achievement to enhance the attainment of the LDL-C goals.

## 2. Materials and Methods

This was an observational, retrospective, single-center study of 271 patients who began treatment with PCSK9-mAb at the Center for Atherothrombotic Diseases of Careggi University Hospital between April 2018 and December 2022. Patients received evolocumab at a dose of 140 mg every 2 weeks, or alirocumab, at doses of 75 mg or 150 mg, every 2 weeks (delivered via a single pre-filled syringe for subcutaneous injection in the abdomen, thigh, or upper arm).

PCSK9mAb treatments were prescribed according to the ESC Guidelines’ recommendations and the Italian Medicines Agency (AIFA) reimbursement criteria. Specifically, patients aged 18 to 80 years old received PCSK9mAb treatment if they had:

1. Asymptomatic HeFH with LDL-C levels ≥ 130 mg/dL despite at least 6 months of treatment with a high-intensity statin at the highest tolerated dose in combination with ezetimibe, or with demonstrated statin and/or ezetimibe intolerance;
2. Known ASCVD with LDL-C levels ≥ 70 mg/dL (≥100 mg/dL until 15 June 2022) despite at least 6 months of treatment with a high-intensity statin at the highest tolerated dose in combination with ezetimibe or with demonstrated statin and/or ezetimibe intolerance or after a single LDL-C detection in cases of recent acute myocardial infarction (within the last 12 months) or multiple cardiovascular events;
3. Diabetes mellitus (DM) with target organ damage or an additional major risk factor and LDL-C levels ≥ 70 mg/dL despite at least 6 months of treatment with a high-intensity statin at the highest tolerated dose in combination with ezetimibe, or with demonstrated statin and/or ezetimibe intolerance.

HeFH was diagnosed clinically using Dutch Lipid Clinical Network Score for FH (DLCNS), with or without genetic analysis.

All patients underwent a clinical evaluation at the initiation of PCSK9mAb therapy, which included demographics, cardiovascular risk factors, history of ASCVD, dyslipidemia type, comorbidities, type and dose of PCSK9mAb and other lipid lowering therapies (LLTs), such as statins, ezetimibe, and statins plus ezetimibe. Statin and ezetimibe intolerance was determined through patient-reported symptoms and/or through laboratory test results.

The lipid profile was assessed before starting PCSK9mAb therapy and during follow-up at 6, 12, and 24 months. Patients were included in the final analysis if they had at least one post-baseline LDL-C value.

Changes in LLT, adverse drug reactions (ADRs), and major adverse cardiac and cerebrovascular events (MACCE) were collected. Data were recorded by investigators using electronic health records.

The primary outcome of interest in this study was patients’ adherence to the 2019 ESC Guidelines’ recommendations on dyslipidemias [7]. Therefore, we assessed the proportion of patients achieving an LDL-C reduction of ≥50% from baseline plus their CV risk class-specific LDL-C targets: <55 mg/dL for patients with known ASCVD and/or DM with target organ damage or an additional major risk factor (very-high-risk CV patients) and <70 mg/dL for patients with asymptomatic HeFH (high-risk CV patients). Furthermore, we searched for possible predictors of target attainment, with a focus on the role of background oral LLT.

### Staistical Analysis

Statistical analysis was performed using IBM SPSS Statistics for Macintosh, version 27.0 (IBM Corp, Armonk, NY, USA). Data were described as frequencies for categorical variables, mean values with standard deviations for normally distributed continuous data, and as medians [interquartile range (Q1, Q3)] for non-normally distributed continuous data. The unpaired t test or Wilcoxon rank-sum test was used to compare statistically significant differences between groups for the continuous variables. The chi-squared test was performed to analyze categorical variables. Statistical significance was defined as *p* < 0.05. A multivariate Poisson regression model was used to detect the possible predictors of LDL-C target achievement. The analysis was stratified into high CV risk and very high CV risk.

## 3. Results

### 3.1. Baseline Characteristics

Between April 2018 and December 2022, therapy with PCSK9mAb was prescribed to 271 patients. Among these, 256 (94.5%) had at least one post-baseline LDL-C measurement and were included in the final analysis.

The demographic and baseline clinical characteristics of the 256 patients are listed in Table 1.

#### 3.1.1. High-CV-Risk Patients

Fifty-three patients were included in this category, all with a diagnosis of HeFH without ASCVD. The median age was 59.02 ± 13.56 years, and 33 (62.3%) were female. The median LDL-C at baseline was 185.27 ± 45.45 mg/dL.

Forty patients (75.5%) were intolerant to statins. Fifteen (28.3%) patients were intolerant to either statins or ezetimibe and were not taking any LLTs.

Regarding PCSK9mAb prescription, 64.1% of the patients (*n* = 34) started on alirocumab, mostly at the higher dosage of 150 mg (*n* = 28, 52.8%).

#### 3.1.2. Very-High-Risk Patients

All patients included in the very-high-CV-risk category (*n* = 203) had a known ASCVD. Among these subjects, 79 (38.9%) also had a diagnosis of HeFH. The median age was 67 ± 11 years; 62 (30.5%) were female.

The most frequent diagnosis was coronary artery disease, primarily presenting as acute myocardial infarction (*n* = 100, 39.1%).

At baseline, most of this cohort was treated with a statin plus ezetimibe (*n* = 102, 50.2%). Among them, 82 (80.4%) received a high-intensity statin. Statin intolerance was reported in 106 (52.2%) cases.

More than half of the patients (*n* = 118, 58.1%) started on evolocumab at a dosage of 140 mg.

### 3.2. Follow-Up: Efficacy

#### 3.2.1. High-CV-Risk Patients

After one year of treatment, average baseline LDL-C levels were significantly reduced from 185.27 ± 45.45 to 82.73 ± 41.15 (Table 2).

**Figure 1:**
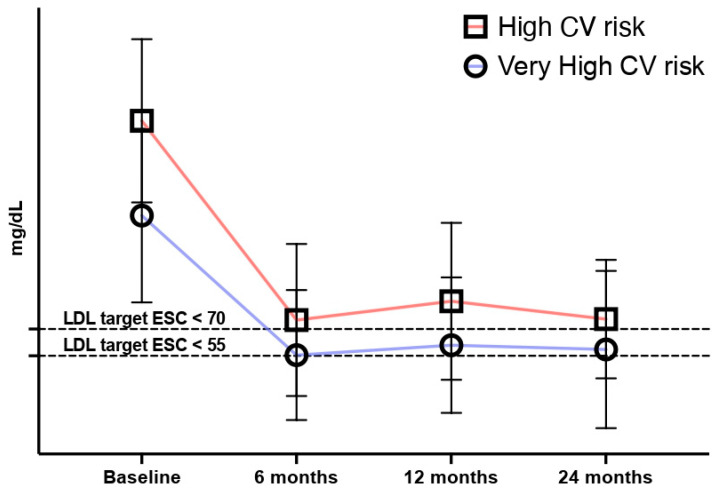
Median LDL-C levels. The graph and the subsequent statistical analysis show a statistically significant reduction in the mean values of LDL-C between baseline and follow-ups (6; 12; 24 months). However, there is no substantial and statistically significant variation between the values at different follow-ups, with mean values slightly above the target set by the ESC recommendations.

**Figure 2:**
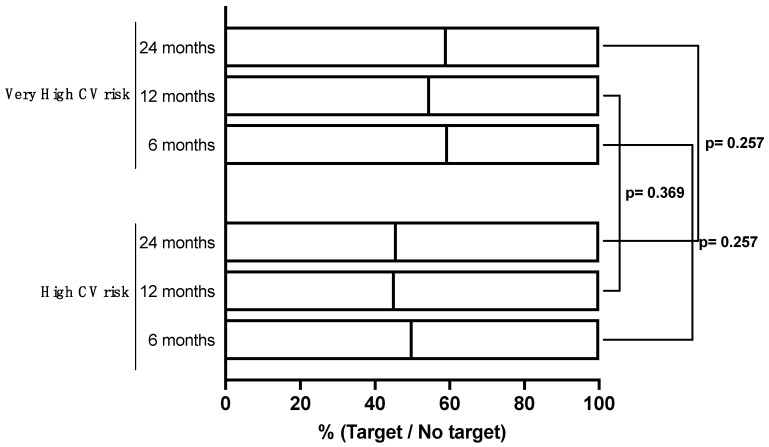
LDL-C target achievement stratified by CV risk class (≥50% LDL-C reduction PLUS LDL-C < 55 or <70 mg/dL, respectively).

Thirty-six patients (67.9%) experienced a reduction of at least 50% in their LDL-C values and 22 (41.5%) showed full adherence to the ESC Guidelines’ recommendations (at least a 50% reduction in LDL-C levels plus LDL-C target achievement). These rates did not change significantly at subsequent follow-up, where available (Table 3, Figure 1 and Figure 2).

Focusing on the 31 patients (58.4%) who did not reach the recommended target at 12 months, 12 patients (38.7%) started bempedoic acid treatment from March 2023. Of these, we have 3-month follow-up data available for nine (75%) patients: three (33.3%) reached the recommended target.

#### 3.2.2. Very-High-Risk Patients

After one year of treatment, average baseline LDL-C levels were significantly reduced from 133.21 ± 48.73 to 54.74 ± 34.70 (Table 2).

Overall, 128 patients (66.7%) experienced a reduction of at least 50% in their LDL-C values and 104 (54.2%) showed full adherence to the ESC Guidelines’ recommendations (at least a 50% reduction in LDL-C levels plus LDL-C target achievement). These rates did not change significantly at subsequent follow-up (Table 3, Figure 1 and Figure 2).

Focusing on the 88 patients (45.8%) who did not reach the recommended target at 12 months, 23 patients (26.1%) started bempedoic acid treatment from March 2023. For these patients, we have the 3-month follow-up data available for 16 (69.6%) patients, and 9 (56.3%) reached the recommended target.

### 3.3. Predictors of LDL-C Target Achievement

We performed an analysis to identify possible predictors of LDL-C target achievement at 12 months. Table 4 and Table 5 show the results of univariable and multivariable Poisson regression analysis for LDL-C target level attainment in patients at high and very high CV risk, respectively.

#### 3.3.1. High-CV-Risk Patients

After adjusting for CV risk factors, comorbidities and LLTs therapy, background therapy with at least one LLTs was the only independent variable associated with a higher probability of achieving LDL-C target levels. Specifically, 18 (58%) patients with at least one LLTs achieved the LDL-C target while only 6 (24%) patients without at least one LLTs reached the target (*p* = 0.014) (median LDL-C 59.40 ± 38.75 mg/dL vs. 74.50 ± 37.32 mg/dL, *p* < 0.007)

#### 3.3.2. Very-High-Risk Patients

After adjusting for CV risk factors, comorbidities, and LLTs, we identified hypertension and high-intensity statin therapy as independent variables associated with the probability of achieving the LDL-C target levels. After one year, lower median LDL-C levels were recorded in patients treated with an high-intensity statin (46.84 ± 35.44 mg/dL), especially in combination with ezetimibe (46.32 ± 28.68). This combination therapy resulted in a higher probability of reaching the LDL-C target level (70.7% vs. 51.8%, *p* = 0.024).

On the other hand, statin intolerance, HeFH, and female gender were associated with a significant lower probability of achieving LDL-C target levels. Forty-four (44%) patients with statin intolerance achieved the LDL-C target compared to 60 (66.7%) patients without statin intolerance (*p* = 0.003). Patients with statin intolerance presented with higher median LDL-C values than patients who could take statins (66.51 ± 36.757 mg/dL vs. 50.85 ± 38.261, *p* = 0.001).

Only 35 patients (47%) with HeFH achieved the LDL-C target, compared to 70 patients (60.3%) without HeFH (*p* = 0.078). Patients with HeFH, as expected, presented with higher median LDL-C values (73.56 ± 41.63 mg/dL vs. 52.24 ± 32.19, *p* < 0.001). Regarding gender, 83 male (60%) achieved the target compared to 23 females (41.8%) (*p* = 0.017). LDL-c median levels were significantly higher in females compared to males (73.52 ± 38.46 vs. 51.6 ± 35.64 mg/dL; *p* < 0.001).

### 3.4. Safety

#### 3.4.1. High-CV-Risk Patients

Four patients (7.5%) reported mild injection-site reactions; however, none of them discontinued the treatment. One patient (1.9%) experienced flu-like symptoms in the days following drug administration, one patient (1.9%) reported skin rashes, and one patient (1.9%) had osteo-articular manifestations (myalgia and athralgia). Three patients (5.7%) switched to inclisiran therapy, one (1.9%) due to side effects (flu-like symptoms), and two (3.8%) due to low therapeutic adherence. The remaining patients demonstrated a 100% adherence.

#### 3.4.2. Very-High-Risk Patients

Nine patients (4.4%) reported mild injection-site reactions; however, none of them discontinued the treatment. Two patients (0.9%) experienced flu-like symptoms in the days following drug administration, two patients (0.9%) reported skin rashes, and one patient (0.9%) had osteo-articular manifestations. Only five patients (2.4%) switched to inclisiran therapy: two (0.9%) due to side effects (one for flu-like symptoms and one for skin rashes), and three (1.5%) due to low therapeutic adherence.

## 4. Discussion

In this real-world analysis, our aim was to evaluate the effectiveness and safety of PCSK9mAb in routine clinical practice. Although the efficacy of this novel drug class is well established in improving adherence to LDL-C targets, continuous clinical surveillance is necessary to enhance adherence to guideline recommendations in the real world.

We report the main results of this study:

- In our cohort, which included patients at high and very high cardiovascular risk, nearly 60% of patients demonstrated full adherence to the ESC Guidelines’ recommendations after one year of PCSK9mAb treatment. This was defined as achieving at least a 50% reduction in LDL-C levels along with reaching the LDL-C target levels based on the cardiovascular risk classification (≤55 and ≤70 mg/dL respectively).
- A background oral LLT involving a high-dose statin emerged as the primary predictor of LDL-C target attainment.
- HeFH and statin intolerance were associated with a significantly lower probability of achieving LDL-C target levels. Unexpectedly, female gender also emerged as a negative predictive factor for a favorable treatment response.
- We reaffirmed the high levels of adherence to these treatments in a real-world setting.

Over the years, recommended target LDL-C levels have progressively decreased, and we currently have very ambitious targets along with new therapies capable of achieving them [6,7]. Nevertheless, in clinical practice, patients often remain undertreated, resulting in suboptimal achievement of the LDL-C targets. The EUROASPIRE V survey, which collected information on 7824 patients from 130 centers in 27 countries, clearly shows that the overall lipid control was unsatisfactory in a large proportion of patients, with fewer than 30% of individuals achieving LDL-C levels at or below 70 mg/dL [11]. The DA VINCI study, a cross-sectional study enrolling 5888 patients, showed that fewer than half of high-/very-high-CV-risk patients achieved the 2016 ESC LDL-C goals [8]. Goal attainment further declined with the implementation of the more stringent 2019 ESC Guidelines. The SANTORINI study, a multinational, multicenter, prospective observational non-interventional study recruiting 9606 patients with high and very high CV risk requiring LLT from different European countries and care settings, showed that only 20.7% of the very high-risk patients reached their LDL-C recommended target [19].

These findings have been confirmed by other real-world registries [17,18,24]. HEYMANS was a prospective registry of 1951 adults initiating evolocumab in routine clinical practice in 12 European countries after August 1 2015. In this registry, almost 60% of patients at (very) high CV risk achieved the goal of an LDL-C level of less than 55 or 70 mg/dL according to the 2019 ESC guidelines, with approximately 60% achieving at least a 50% reduction from baseline [17]. A Spanish study, which enrolled patients who started on alirocumab or evolocumab from February 2017 to April 2020, found a median reduction of 59.9% in LDL-C values with these drugs, with almost 50% of patients fulfilling the 2019 ESC recommendations [25]. Similar results were shown by AT-TARGET-IT, an Italian multicenter observational registry of 798 patients treated with PCSK9mAb at 10 Italian sites between 2017 and 2021.

The results of our analysis are consistent with these data. Indeed, we recorded an overall median reduction of 57.83 ± 24.5% in LDL-C levels after PCSK9mAb initiation, which was maintained throughout the study period [14,15,26]. This relative reduction led almost 60% of our population to fulfill the 2019 ESC Guidelines’ recommendations, without significant differences between CV risk categories and the two PCSK9mAb molecules.

However, based on clinical trial results, we expected a higher percentage of LDL-C target attainment. Indeed, a systematic literature review and network meta-analysis conducted on randomized controlled trials of non-statin LLTs added to maximally tolerated statins, including statin-intolerant patients, showed that a proportion greater than 70% of LDL-C values achieved the 2019 ESC Guideline goal with either evolocumab at a dose of 140 mg or alirocumab at a dose of 150 mg [27]. This discrepancy with real-world data could have two possible explanations. On one hand, clinical trials are based on a selected patient population that, although similar, does not exactly reproduce everyday patients. Furthermore, clinical trials are conducted according to a specific protocol that does not allow the variations that are part of normal clinical practice. On the other hand, a smaller percentage of oral LLTs used in the real world could also affect this result. For example, in the FOURIER trial, 70% of patients took a high-intensity statin vs. almost 40% of our real-world population, and almost 99.6% of all the patients were taking at least a low-intensity statin. Similarly, in the ODISSEY trial, 47% of patients took a high-intensity statin, with only 2.5% of patients being statin-intolerant and not taking any background LLTs.

Indeed, one of the main reasons for not achieving the target in our study was LLT underuse and statin intolerance. The absence of concomitant oral lipid-lowering therapy, especially a high-intensity statin, played a key role among the predictors of therapeutic failure.

Previous studies have shown that adherence to and persistence with statins are low. The consequential undertreatment can adversely affect clinical outcomes [28]. Despite solid clinical evidence and guideline recommendations, statins are still underused and often underdosed. Recent data showed that statins are prescribed in approximately only 50% of patients with ASCVD, 55% of patients with coronary artery disease, and 40% of patients with peripheral arterial disease [29,30]. Barriers to statin therapy implementation can be attributed to both patients and physicians.

Most patients dislike statin therapy due to a poor perception of hypercholesterolemia and the fear of myalgia, which is not always, but often, a nocebo effect. On the other hand, physicians lack adequate follow-up plans and often exhibit clinical inertia, leading to suboptimal LDL-C management [31]. In our population, the main barrier to background therapy implementation was the high percentage of statin intolerance, especially in the primary prevention subgroup (75.5% vs. 52.2%, *p* = 0.002). This can be explained by the nature of our center, which predominantly receives patients who do not tolerate first-line LLTs.

The development of PCSK9mAb has provided a greater opportunity to achieve more effective lipid control in high-cardiovascular-risk patients and to overcome intolerance issues, allowing for a LDL-C reduction of up to 60% [14,15,26]. Nevertheless, real-world data reported that a greater use of combination therapy is required for patients to achieve the ESC/EAS LDL-C goals.

The HEYMANS study highlighted that more patients receiving evolocumab in combination with LLT attained their ESC/EAS LDL-C goals than those not receiving combination therapy [17].

In AT-TARGET-IT, 83.3% of patients achieving the LDL-C target were on a combination therapy with PCSK9mAb and background LLT (mostly a single-pill combination of a statin with ezetimibe) [18]. Thus, statins cannot be regarded as an outdated therapy but as the foundation on which the treatment of cardiovascular prevention (primary and secondary) is built.

Another factor influencing target achievement was HeFH, with fewer than 50% of very-high-CV-risk patients with HeFH reaching their LDL-C target.

Recent real-world studies have underscored that LDL-C levels often remain elevated in the majority of patients with HeFH, despite conventional LLT. For instance, a retrospective observational study covering the 4-year use of PCSK9mAb in Italy after their approval in 2017 revealed that, despite substantial reductions in LDL-C, only 42% of patients with HeFH treated with PCSK9mAb met the ESC Guidelines’ recommendations. This study attributed this limited efficacy of PCSK9mAb in achieving LDL-C targets in many patients with HeFH to their initially high LDL-C levels at treatment initiation [32]. Similarly, in our HeFH patients, the lower LDL-C target achievement appears to be primarily linked to the higher LDL-C levels observed. A recent cross-sectional observational study conducted across three academic centers in the Netherlands and Norway revealed that only a minority (27.7%) of familial hypercholesterolemia patients reached the guideline-recommended LDL-C treatment target [33]. We did not find disparities in adherence to PCSK9mAb between patients with HeFH and those with non-familial hypercholesterolemia, and HeFH status remained independent of background oral LLT for target achievement.

Unexpectedly, female gender emerged as another independent variable associated with a lower likelihood of achieving target LDL-C levels. This finding aligns with prior research indicating a gender disparity in dyslipidemia management, where women are significantly more likely to fail to meet LDL-C targets and less likely to receive high-intensity lipid lowering medications in response to elevated LDL-C levels. Indeed, despite increased awareness of cardiovascular risk in women and growing research on gender-based treatment disparities, inequities persist in medical management [12,34,35,36,37]. The underlying reasons for the differential approach to LDL-C management between genders warrant further investigation, although some potential explanations have been identified. Firstly, there is a known underestimation of cardiovascular risk among female patients and their clinicians, along with hesitancy to prescribe statins to women, often due to misconceptions that women are inherently protected against atherosclerotic cardiovascular diseases [34,35,38]. Moreover, studies have shown a stronger association between female sex and adverse drug reactions to lipid-lowering therapies, such as statin-associated muscle symptoms (SAMS), leading to less aggressive treatment by physicians [39,40,41,42,43]. However, in our registry, female gender was found to be independent of statin intolerance and background oral lipid-lowering therapy for target achievement, with no significant differences observed in statin tolerance between sexes. A recent real-world registry study revealed that the addition of PCSK9mAb to maximum tolerated lipid-lowering therapy resulted in a relatively smaller reduction in LDL-C levels among women compared to among men at 6-month follow-up, not only linked to the higher baseline LDL-C levels in women. The unequal reduction in LDL-C between the two sexes is partially explained by the presence of higher circulating levels of PCSK9 in women, especially post-menopause, resulting in higher levels of LDL-C. In our cohort, females had higher LDL-C baseline levels than males, primarily due to the higher rates of HeFH in females, while a relatively smaller reduction in LDL-C levels was not observed. Nonetheless, further research is necessary to elucidate potential hormone-related sex differences in LDL-C response to PCSK9mAb. This hormonal influence could offer a plausible explanation for our findings.

Our analysis confirms the favorable safety profile of PCSK9mAb. Only 8.2% of our patients reported adverse drug reactions, mostly mild and transient.

### Study Limitations

The main limitations of this study are the single-center evaluation, and the relatively small number of patients enrolled. Additionally, due to the observational design, potential misclassifications of data may have occurred.

We acknowledge the possibility of referral bias, as most patients were referred to our center due to suspected HeFH and a high level of statin intolerance. Furthermore, our center’s extensive experience in dyslipidemia management may limit the generalizability of these observations to other settings.

## 5. Conclusions

In conclusion, our analysis confirms that PCSK9mAb treatment is safe and effective, enabling most patients in our cohort to reach their LDL-C targets. The concurrent use of oral lipid-lowering therapy, particularly high-intensity statins, emerged as a crucial factor, significantly affecting therapeutic efficacy. Thus, our findings underscore the importance of combination therapy in effectively managing patients at (very) high cardiovascular risk, and the need for its persistence over time. Additionally, familial hypercholesterolemia and female gender were identified as predictors of therapeutic failure. It is therefore crucial to address disparities in cardiovascular disease prevention between genders and to enhance strategies for managing elevated LDL-C in HeFH patients. These steps are essential for ensuring equitable access to effective treatment and improving outcomes for all patients at risk of cardiovascular disease.

## Figures and Tables

**Table 1:** Demographical and baseline clinical characteristics.

Variables		Overall *n* = 256	High Risk, *n* = 53	Very High Risk, *n* = 203	*p* *
Demographical characteristics	Women, *n* (%)	95 (37.1)	33 (62.3)	62 (30.5)	<0.001 *
	Age (years old), mean ± DS	65.43 ± 11.12	59.12 ± 13.65	67.11 ± 9.67	<0.001 *
Cardiovascular risk factors	Smokers, *n* (%)	28 (10.9)	6 (11.3)	22 (10.8)	0.920
	Ex smokers, *n* (%)	114 (44.5)	15 (28.3)	99 (48.8)	0.008 *
	Hypertension, *n* (%)	160 (62.5)	19 (35.8)	140 (69)	<0.001 *
	T2DM, *n* (%)	31 (12.1)	0 (0)	31 (15.3)	0.002 *
	Family history of ASCVD, *n* (%)	118 (46.3)	22 (41.5)	96 (47.5)	0.435
	Hyperuricemia, *n* (%)	5 (2)	0 (0)	5 (2.5)	0.247
CAD	AMI, *n* (%)	100 (39.1)	/	100 (49.3)	/
	Unstable angina, *n* (%)	26 (10.2)	/	26 (12.8)	/
	Stable angina, *n* (%)	68 (26.6)	/	68 (33.5)	/
	PCI, *n* (%)	141 (55.1)	/	141 (69.5)	/
	CABG, *n* (%)	27 (10)	/	27 (13.3)	/
Others ASCVD	Stroke/TIA, *n* (%)	20 (7.8)	/	20 (9.9)	/
	Carotid disease, *n* (%)	18 (7)	/	18 (8.9)	/
	PAD, *n* (%)	35 (13.7)	/	35 (17.2)	/
	Carotid revascularization, *n* (%)	19 (7.4)	/	19 (9.4)	/
	Peripheral revascularization, *n* (%)	11 (4.3)	/	11 (5.4)	/
Comorbidities	Heart failure, *n* (%)	10 (3.9)	1 (1.9)	9 (4.4)	0.395
	Chronic kidney disease, *n* (%)	5 (2)	0 (0)	5 (2.5)	0.249
Dyslipidemia type	HeFH, *n* (%)	132 (51.6)	53 (100)	76 (37.4)	<0.001 *
	Non-familial hypercholesterolemia, *n* (%)	93 (36.3)	0 (0)	93 (45.8)	<0.001 *
	Mixed Dyslipidemia, *n* (%)	31 (12.1)	0 (0)	31 (15.3)	0.002 *
LLTs	None, *n* (%)	40 (15.6)	15 (28.3)	25 (12.3)	0.004 *
	High dose statin alone, *n* (%)	10 (3.9)	0 (0)	10 (4.9)	0.100
	Moderate dose statin alone, *n* (%)	2 (0.8)	0 (0)	2 (1)	0.469
	Low dose statin alone, *n* (%)	0 (0)	0 (0)	0 (0)	1.000
	Ezetimibe alone, *n* (%)	88 (34.4)	24 (45.3)	64 (31.5)	0.061
	High dose statin + ezetimibe, *n* (%)	91 (35.5)	9 (17)	82 (40.4)	0.002 *
	Moderate dose statin + ezetimibe, *n* (%)	22 (8.6)	4 (7.5)	18 (8.9)	0.761
	Low dose statin + ezetimibe, *n* (%)	3 (1.2)	1 (1.9)	2 (1)	0.588
	Statin Intolerance, *n* (%)	146 (57)	40 (75.5)	106 (52.2)	0.002 *
PCSK9i type and dose	Repatha 140 mg, *n* (%)	137 (53.5)	19 (35.8)	118 (58.1)	0.004 *
	Praluent 150 mg, *n* (%)	97 (37.9)	28 (52.8)	69 (34)	0.012 *
	Praluent 75 mg, *n* (%)	23 (9)	6 (11.3)	17 (8.4)	0.505

DS: standard deviation; T2DM: type 2 diabetes mellitus; ASCVD: atherosclerotic cardiovascular disease; CAD: coronary artery disease; AMI: acute myocardial infarction; PCI: percutaneous Coronary intervention: CABG: coronary artery bypass graft surgery; TIA: transient ischemic attack; PAD: peripheral artery disease; HeFH: heterozygous familial hypercholesterolemia; LLTs: lipid-lowering therapies; PCSK9i: proprotein convertase subtilisin/kexin type 9 inhibitor. *: *p* < 0.05—(high CV risk vs. very high CV risk).

**Table 2:** LDL-C levels during follow-up.

Visit	Overall, Mean ± DS	High CV Risk, Median ± DS	Very High CV Risk, Median ± DS	*p*
Baseline	144.59 ± 52.58	185.27 ± 45.45	133.21 ± 48.73	<0.001 *
6 month	59.57 ± 37.56	76.79 ± 42.38	54.74 ± 34.7	<0.001 *
12 month	63.47 ± 38.88	82.73 ± 41.15	57.89 ± 36.44	<0.001 *
24 month	61.38 ± 41.76	77.21± 33.32	57.5 ± 42.83	0.002 *

*: *p* < 0.05—(high CV risk vs. very high CV risk).

**Table 3:** LDL-C target achievement.

LDL-C Goal of <1.4 mmol/L (<55 mg/dL)				
Visit	Overall, *n* (%)	High CV Risk, *n* (%)	Very High CV Risk, *n* (%)	*p*
6 month (*n* = 256)	147 (57.4%)	28 (50%)	119 (59.5%)	0.204
12 month (*n* = 245)	129 (52.7%)	24 (45.3%)	105 (54.7%)	0.128
24 month (*n* = 122)	69 (56.6%)	11 (45.8%)	58 (59.2%)	0.237
≥50% LDL-C reduction from baseline				
6 month (*n* = 256)	178 (69.5%)	42 (79.2%)	136 (67.0%)	0.314
12 month (*n* = 245)	164 (66.9%)	36 (67.9)	128 (66.7%)	0.790
24 month (*n* = 122)	89 (72.9%)	16 (66.7%)	73 (74.5%)	0.439
≥50% LDL-C reduction from baseline and an LDL-C goal of <1.4 mmol/L (<55 mg/dL)				
6 month (*n* = 256)	144 (56.3%)	28 (52.8%)	116 (57.1%)	0.286
12 month (*n* = 245)	126 (51.5%)	22 (41.5%)	104 (54.2%)	0.054
24 month (*n* = 122)	68 (55.8%)	11 (45.8%)	57 (58.2%)	0.276

**Table 4:** Univariable regression analysis for LDL-C target achievement (≥50% LDL-C reduction PLUS LDL-C < 55 or <70 mg/dL).

	Very High CV Risk			High CV Risk		
Variables	IRR	95% C.I.	*p*	IRR	95% C.I.	*p*
Female gender	0.465	0.25–0.88	0.018 *	0.667	0.22–2.02	0.473
Smokers	0.968	0.39–2.36	0.943	2.9	0.48–17.38	0.244
Ex-smokers	1.361	0.77–2.42	0.293	1.184	0.36–3.9	0.782
Hypertension	1.833	0.98–3.4	0.055	1.538	0.52–4.57	0.438
T2DM	1.631	0.69–3.87	0.267	0.000	0	0.999
Family history of ASCVD	0.805	0.45–1.43	0.460	1.385	0.47–4.05	0.552
CAD	2.543	1.17–5.53	0.019 *	/	/	/
Stroke/TIA	1.033	0.4–2.65	0.652	/	/	/
PAD	0.895	0.46–1.74	0.649	/	/	/
Peripheral revascularization	2.343	0.89–6.16	0.052	/	/	/
HEFH	0.691	0.39–1.23	0.078	/	/	/
Non-familial hypercholesterolemia	1.383	0.78–2.45	0.168	/	/	/
Mixed Dyslipidemia	0.932	0.43–2.03	0.817	/	/	/
Statin Intolerance	0.409	0.23–0.74	0.003 *	0.321	0.09–1.135	0.078
At least one LLT	2.157	1.08–4.34	0.031 *	4.154	1.29–13.35	0.017 *
Statin + ezetimibe	2.102	1.14–3.89	0.018 *	3.111	0.68–14.04	0.140
High dose statin	2.890	1.41–5.91	0.004 *	7.895	0.86–72.88	0.068
High dose statin + ezetimibe	2.321	1.1–4.89	0.027 *	0.221	0.42–44.1	0.221

*: *p* < 0.05 (Univariable regression analysis).

**Table 5:** Multivariable regression analysis for LDL-C target level achievement (≥50% LDL-C reduction PLUS LDL-C < 55 or <70 mg/dL).

	Very High CV Risk			High CV Risk		
Variables	IRR	95% C.I.	*p*	IRR	95% C.I.	*p*
Female gender	0.473	0.24–0.94	0.033 *	/	/	/
Hypertension	2.025	1–4.1	0.050 *	/	/	/
HEFH	0.484	0.25–0.96	0.037 *	/	/	/
Statin Intolerance	0.484	0.23–1.01	0.055 *	/	/	/
At least one LLT	/	/	/	4.154	1.29–12.35	0.013 *
High dose statin	3.244	1.27–8.27	0.014 *	/	/	/

*: *p* < 0.05 (Multivariable regression analysis).

## Data Availability

The data presented in this study are available on request from the corresponding author due to privacy restrictions. As this study involves human patients, the original data source contains private patient information. Disclosure of these data requires authorization from the data protection authority and anonymization of the databases.
